# Assessment of MRI Issues at 3 Tesla for a New Metallic Tissue Marker

**DOI:** 10.1155/2015/823759

**Published:** 2015-07-22

**Authors:** Charlotte M. Cronenweth, Frank G. Shellock

**Affiliations:** ^1^Loyola Marymount University, 1 LMU Drive, Los Angeles, CA 90045, USA; ^2^Keck School of Medicine, University of Southern California and Institute for Magnetic Resonance Safety, Education, and Research, 7751 Veragua Drive, Playa Del Rey, CA 90293, USA

## Abstract

*Purpose*. To assess the MRI issues at 3 Tesla for a metallic tissue marker used to localize removal areas of tissue abnormalities. *Materials and Methods*. A newly designed, metallic tissue marker (Achieve Marker, CareFusion, Vernon Hills, IL) used to mark biopsy sites, particularly in breasts, was assessed for MRI issues which included standardized tests to determine magnetic field interactions (i.e., translational attraction and torque), MRI-related heating, and artifacts at 3 Tesla. Temperature changes were determined for the marker using a gelled-saline-filled phantom. MRI was performed at a relatively high specific absorption rate (whole body averaged SAR, 2.9-W/kg). MRI artifacts were evaluated using T1-weighted, spin echo and gradient echo pulse sequences. *Results*. The marker displayed minimal magnetic field interactions (2-degree deflection angle and no torque). MRI-related heating was only 0.1°C above background heating (i.e., the heating without the tissue marker present). Artifacts seen as localized signal loss were relatively small in relation to the size and shape of the marker. *Conclusions*. Based on the findings, the new metallic tissue marker is acceptable or “MR Conditional” (using current labeling terminology) for a patient undergoing an MRI procedure at 3 Tesla or less.

## 1. Introduction

With an increased use of magnetic resonance imaging (MRI) for characterization of abnormal tissue, especially with regard to breast lesions, there is a concomitant demand for tissue markers that are “visible” and relatively harmless in this setting. Tissue markers are commonly used in association with biopsy procedures to mark the location in soft tissue for future surgical procedures. For example, markers can be used to localize a tumor and allow for the monitoring of growth or recurrence of a lesion [1–3]. Specifically, breast biopsy tissue markers are generally used for tumor localization for subsequent tumor resection [2, 3].

For tissue markers made from metal, there are possible issues related to performing MRI procedures in patients with these implants, particularly with the use of high-field-strength (i.e., 3 Tesla) MR systems [4, 5]. Therefore, the purpose of this investigation was to assess a newly developed, metallic tissue marker using standardized testing techniques to characterize magnetic field interactions, MRI-related heating, and artifacts at 3 Tesla. These testing procedures are vital to ensure patient safety in the MRI setting when a metallic implant is present [4, 5].

## 2. Materials and Methods

### 2.1. Tissue Marker

**Figure 1:**
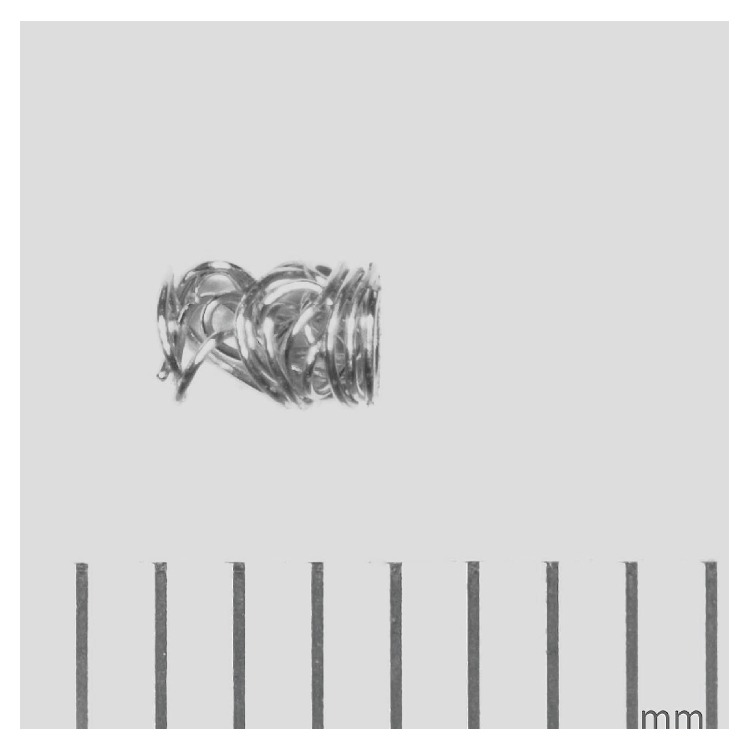
The metallic tissue marker (Achieve Marker, 3-mm length, CareFusion, Vernon Hills, IL) that underwent testing for MRI issues at 3 Tesla. Note the irregular surface, allowing for growth of tissue in and around this implant to reduce migration once it is deployed.

A newly developed, metallic tissue marker (Achieve Marker, 3-mm length; 12-gauge, 99.9% gold material, CareFusion, Vernon Hills, IL) (Figure 1) was selected for assessment of MRI issues at 3 Tesla. The features of this marker include that it is the same size as the biopsy that is obtained and has a configuration (i.e., an irregular surface allowing for growth of tissue around it) that is specially designed to prevent migration once it is deployed in tissue. The marker is supplied in a delivery device that approximates the same size of the biopsy device (Achieve Biopsy Device, CareFusion, Vernon Hills, IL), thus, permitting precise placement.

### 2.2. Magnetic Field Interactions

#### 2.2.1. Translational Attraction

Translational attraction was determined at 3 Tesla for the tissue marker using the deflection angle method, as previously described [6–13]. The marker was suspended from a 20-cm length of light-weight string (less than 10% of the weight of the marker) that was attached at the 0-degree position on a protractor that was fixed on a test apparatus [6–13]. The apparatus was positioned in a 3 Tesla MR system (Excite, HDx, Software 14X.M5, General Electric Healthcare, Milwaukee, WI) at the point of the highest “patient accessible” spatial gradient magnetic field for the 3-T MR system [6–13]. The value of the spatial gradient magnetic field at this point was determined using a gaussmeter (Extech 480823 Electromagnetic Field and Extremely Low Frequency Meter; Extech, Nashua, NH) and was found to be 720 gauss/cm, occurring at an off-axis position, 74-cm from isocenter of the MR system [7–13]. The deflection angle from the vertical position to the nearest 1 degree was measured three times, and the mean value was calculated [6–13].

#### 2.2.2. Torque

Magnetic field-induced torque was determined qualitatively for the tissue marker using a standardized test, as previously described [7–10, 12]. This test consisted of placing the marker on a flat plastic grid with millimeter markings at a 45-degree orientation relative to the direction of the 3 Tesla static magnetic field. The apparatus was then placed at the center of the MR system, where the effect of torque on metallic objects is known to be the greatest [7–10, 12]. Movement of the marker with respect to rotation relative to the static magnetic field was carefully observed and this procedure was repeated three times to encompass full 360 degrees of rotation for the implant. A mean value of torque was calculated for the marker with it being oriented along its short and long axes, according to the following scale: 0: no torque; +1: mild or low torque, the implant moved slightly but did not align with the magnetic field; +2: moderate torque, the marker eventually aligned with the magnetic field; +3: strong torque, the marker aligned rapidly with the magnetic field; +4: very strong torque, the implant shows very rapid and forceful alignment with the magnetic field [4, 11–15].

### 2.3. Evaluation of MRI-Related Heating

#### 2.3.1. Phantom and Experimental Setup

The tissue marker was assessed for MRI-related heating using a 3 Tesla/128-MHz MR system (Excite, HDx, Software 14X.M5, General Electric Healthcare, Milwaukee, WI). This standardized procedure involved the use of a plastic American Society for Testing and Material (ASTM) International phantom that was filled 10-cm with gelled-saline (i.e., 1.32-g/L NaCl plus 10 g/L polyacrylic acid in distilled water) to simulate human tissue [7–10, 14]. Using this formulation, the room temperature (22°C) electrical conductivity of the gelled-saline was 0.47-S/m. The tissue marker was placed in the phantom at a position known to have the highest uniform electric field tangential to the implant to ensure extreme heating conditions based on an analysis of the ASTM International phantom and the MRI conditions used for this evaluation [7–10, 14]. Because of the lack of perfusion, this testing methodology is an example of extreme MRI-related heating for a metallic implant [7–10, 14].

#### 2.3.2. Temperature Recordings

Temperatures were recorded on the tissue marker using a fluoroptic thermometry system (Model 3100, LumaSense Technologies, Santa Clara, CA) [7–10]. Fluoroptic thermometry probes (Model SFF-2, LumaSense Technologies, Santa Clara, CA) were placed on the ends of the marker to obtain representative temperature recordings insofar as the “ends” of an implant are known to heat the most during MRI-related heating [5, 7–10].

#### 2.3.3. MRI Conditions

MRI was conducted at 3 Tesla using transmit/receive radiofrequency (RF) body coil. The MRI parameters were selected to generate an MR system reported, whole body averaged specific absorption rate (SAR) of 2.9-W/kg. The associated calorimetry value was 2.7-W/kg. The land marking position (i.e., the center position or anatomic region for the MRI) and section locations were selected to encompass the entire tissue marker.

#### 2.3.4. Experimental Protocol

The phantom with the tissue marker was placed in the 3 Tesla MR system and given time to equilibrate for more than 24 hours. The room temperature of the MR system was measured before and after the experiment to confirm that no change greater than 0.2°C occurred during this time. MRI was then performed on the phantom for 15 minutes. Proper fluoroptic thermometry probe positioning relative to the marker was confirmed immediately before and after MRI. The highest temperature changes recorded for the temperature probes attached to the tissue marker are reported, herein.

“Background” temperatures were also recorded in the gelled-saline-filled ASTM International phantom by measuring temperatures in the same fluoroptic thermometry probe positions and at the same time intervals as those used when measuring the temperatures for the tissue marker as part of the MRI-related heating evaluation [7–10, 14]. The highest temperature change obtained from this evaluation is also reported.

### 2.4. Evaluation of Artifacts

Artifacts associated with the metallic tissue marker were evaluated at 3 Tesla. The marker was attached to a plastic frame and placed into a plastic phantom filled with gadolinium-doped, saline, which provided a high signal background [6–10]. MRI was conducted using transmit/receive RF head coil (i.e., for increased signal-to-noise) and the following pulse sequences: T1-weighted, spin echo, repetition time, 500-msec; echo time, 20-msec; matrix size, 256 × 256; section thickness, 10-mm; field of view, 24-cm; number of excitations, 2; bandwidth, 16 kHz, and gradient echo pulse sequence (GRE), repetition time, 100-msec; echo time, 15-msec; flip angle 30°; matrix size, 256 × 256; section thickness, 10-mm; field of view, 24-cm; number of excitations, 2; bandwidth, 16 kHz [6–10]. The imaging planes were positioned to encompass the long axis and short axis of the tissue marker. The image display parameters (i.e., window and level settings, magnification, etc.) were selected and applied in a consistent manner to obtain accurate measurements of the sizes of the artifacts. Planimetry software provided with the MR system was used to measure (accuracy and resolution ±10%) the cross-sectional areas of the largest artifact size for each pulse sequence and imaging plane associated with the tissue marker [6–10]. Notably, this standardized technique of evaluating artifacts has been used in many prior studies that characterized artifacts for metallic implants and, therefore, provides an acceptable means of comparison [5–10].

## 3. Results

Evaluation of magnetic field interactions for the tissue marker yielded a mean deflection angle of 2 ± 0 degrees. There was no torque (i.e., rotational alignment) exhibited by the marker during the qualitative evaluation (i.e., mean torque value, 0, no torque). The MRI-related heating evaluation revealed that the highest change in temperature rise was 1.7°C with the tissue marker present in the phantom. By comparison, the highest temperature rise recorded during the background temperature evaluation was 1.6°C. Therefore, the temperature rise associated with the presence of the metallic tissue marker in the phantom was 0.1°C.

**Figure 2:**
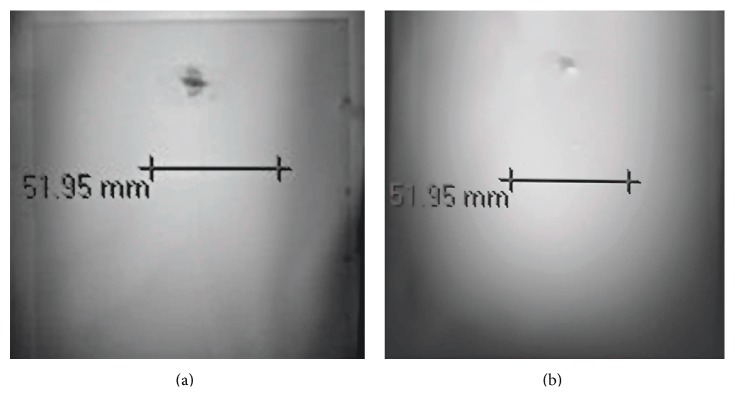
(a) MRI artifact associated with the tissue marker on a gradient echo pulse sequence (TR/TE, 100-msec/15-msec; flip angle, 30 degrees; long axis view; artifact area, 82-mm^2^). The artifact appeared as a low signal intensity signal void that was relatively small in relation to the size and shape of the marker. (b) MRI artifact associated with the tissue marker on a spin echo pulse sequence (TR/TE, 500-msec/20-msec; long axis view; artifact area, 38-mm^2^). The artifact appeared as a low signal intensity signal void that was relatively small in relation to the size and shape of the marker. Note that the artifact size was larger for the gradient echo pulse sequence (a) compared to that observed for the spin echo pulse sequence.

The artifact findings for the tissue marker are summarized in Table 1. Artifacts appeared as low signal intensity signal voids that were relatively small in relation to the size and shape of the marker, with no apparent distortion of the MR images. The GRE pulse sequence (Figure 2(a)) produced larger artifacts than the T1-weighted, spin echo pulse sequence (Figure 2(b)) for the metallic tissue marker.

## 4. Discussion

A tissue marker may be placed in the patient in association with the removal of a tissue sample and acts as a landmark to represent the location of tissue removal for future surgical reference [2, 3, 16]. A biopsy frequently removes the entire lesion; therefore, it has become commonplace to insert a marker so that the site can be easily detected at a later time [2, 3, 15–17].

With regard to markers used for breast tissue, in 2013, it was expected that 232,340 new cases of female invasive breast cancer would be diagnosed in the United States [18]. With the prevalence of stereotactic MRI and ultrasound-guided biopsies accompanying these diagnoses, there is a need for a tissue marker that is permanently visible when using these modalities, that will not migrate, and does not present a risk to patients. Recently, a new tissue marker was developed with these important features and this implant underwent evaluation in the present investigation.

With any metallic implant, concerns arise related to the safety aspects and other potential problems in association with the use of MRI [4, 5]. The standard of care when screening patients prior to performing MRI is to determine if the individual has a metallic implant and, if that is the case, the MRI-specific labeling information must be reviewed (i.e., typically found in the* Instructions for Use* for the product) and carefully followed to ensure patient safety. The labeling information presents the particular parameters that are deemed acceptable for a given implant, according to its “MR Conditional” information (i.e., an item that has been demonstrated to pose no known hazards in a specified MR environment with specific conditions of use indicated) [19]. Importantly, the conditions stated in the labeling are derived from* in vitro* MRI tests that are conducted to assess the MRI issues for the implant [4, 5, 19].

In the present study, a newly developed, metallic tissue marker underwent MRI testing using standardized test procedures in consideration of current clinical MR systems, insofar as 3 Tesla was utilized for the assessment of magnetic field interactions and a relatively high, whole body averaged SAR level was applied during the heating evaluation. While other tissue markers have undergone MRI testing [6, 8, 20–22], to our knowledge, the evaluation of several of these markers did not follow the accepted standardized tests as recommended by the United States Food and Drug Administration (i.e., following the documents from the ASTM International) [23]. Therefore, the relative risks of using MRI in patients with those particular tissue markers are currently unknown.

### 4.1. Magnetic Field Interactions

The metallic tissue marker that underwent evaluation exhibited a 2-degree deflection angle and no torque at 3 Tesla. Thus, there are no risks associated with movement or displacement of this marker in a 3 Tesla or less MRI environment. These results are not surprising because the material (gold) used to make this implant has a low magnetic susceptibility value and, therefore, the associated magnetic field interactions will be minor [5, 24].

### 4.2. MRI-Related Heating

The exposure of a metallic object to MRI has the potential to cause heating under certain conditions [4, 5, 15]. Therefore, as part of proper MRI testing that ensures patient safety, a standardized* in vitro* evaluation is performed to characterize the temperature rises for a metallic implant [4, 5, 15]. The recorded temperature changes for the tissue marker indicated that the highest temperature change was only 0.1°C above the highest background temperature when MRI was conducted at a relatively high whole body averaged SAR level (2.9-W/kg). Obviously, this minimal temperature rise will not pose a risk to the patient with this implant. Although a higher whole body averaged SAR level could have been used (i.e., the current upper limit is an SAR, 4-W/kg), this was not believed to be necessary because the experimental setup involved a nonperfused gelled-saline medium and, thus, created an extreme case for implant heating.

### 4.3. Artifacts

The presence of a metallic implant in a patient undergoing MRI will typically cause low intensity signal loss and, in severe cases, distortion of the image [4, 5, 7–10, 20, 21]. A primary factor that impacts the size of a metal-related artifact is the magnetic susceptibility of the material [24]. The artifacts observed for the tissue marker made from gold (99.99%) appeared as localized signal loss that were relatively small in size in relation to its size and shape (please refer to Figures 2(a) and 2(b)). The gradient echo pulse sequence (i.e., representing an extreme condition for MRI-related artifacts), which is often used for evaluation of the breast or other tissues, produced larger artifacts than the T1-weighted, spin echo pulse sequence.

Notably, an investigation by Genson et al. [20] assessed a range of tissue markers and found that these markers created signal voids that were two to six times the diameter of the marker, which is potentially problematic because subsequent lesion detection may be compromised if the extent of the artifact is too extensive. Because of the low magnetic susceptibility value for gold [24], which is lower than the other materials that have been previously used for metallic markers, this new marker appears to be ideally suited for MRI applications where the extent of the artifact is an important consideration [6, 20–22].

## 5. Conclusions

Findings from MRI tests conducted on a metallic tissue marker indicated that this implant is acceptable or “MR Conditional” (i.e., using current MRI labeling terminology) [19, 24] for a patient undergoing an MRI examination at 3 Tesla or less. This information has significance for screening patients referred for MRI procedures.

Furthermore, the MRI test results for this tissue marker can be applied to other gold markers made from other wire gauges (range 12 to 18 gauges) that have the same or smaller dimensions because the findings will obviously be less than those found for the marker that was tested. This guideline has been applied in prior MRI evaluations of other implants including aneurysm clips, hemostatic clips, and otologic implants that have undergone MRI testing [7, 12, 25].

## Figures and Tables

**Table 1:** Summary of MRI artifacts at 3 Tesla for the tissue marker.

Pulse sequence	T1-SE	T1-SE	GRE	GRE

Signal void size (mm^2^)	38	16	82	61

Imaging plane	Parallel (long axis)	Perpendicular (short axis)	Parallel (long axis)	Perpendicular (short axis)

(T1-SE: T1-weighted, spin echo; GRE: gradient).
